# Health Related Social Needs Among Chinese American Primary Care Patients During the COVID-19 Pandemic: Implications for Cancer Screening and Primary Care

**DOI:** 10.3389/fpubh.2021.674035

**Published:** 2021-05-28

**Authors:** Jennifer Tsui, Annie Yang, Bianca Anuforo, Jolene Chou, Ruth Brogden, Binghong Xu, Joel C. Cantor, Su Wang

**Affiliations:** ^1^Department of Preventive Medicine, Keck School of Medicine, University of Southern California, Los Angeles, CA, United States; ^2^Rutgers New Jersey Medical School, Newark, NJ, United States; ^3^Section of Behavioral Sciences, Rutgers Cancer Institute of New Jersey, New Brunswick, NJ, United States; ^4^Rutgers Center for State Health Policy, Institute for Health, Health Care Policy, and Aging Research, New Brunswick, NJ, United States; ^5^RWJBarnabas Health Medical Group, Center for Asian Health, Saint Barnabas Medical Center, Florham Park, NJ, United States; ^6^Rutgers Edward J. Bloustein School of Planning and Public Policy, New Brunswick, NJ, United States

**Keywords:** social determinants, social needs, cancer screening, Asian American (AA), primary care, COVID-19

## Abstract

**Research Objective:** Initiatives to address social determinants of health (SDOH) and measure health-related social needs (HRSN) within clinic settings are increasing. However, few have focused on the specific needs of Asian Americans (AA). We examine the prevalence of HRSN during a period spanning the COVID-19 pandemic to inform strategies to improve cancer screening and primary care among AA patients.

**Methods:** We implemented a self-administered HRSN screening tool in English and Chinese, traditional (T) or simplified (S) text, within a hospital-affiliated, outpatient primary care practice predominantly serving AA in New Jersey. HRSN items included food insecurity, transportation barriers, utility needs, interpersonal violence, housing instability, immigration history, and neighborhood perceptions on cohesion and trust. We conducted medical chart reviews for a subset of participants to explore the relationship between HRSN and history of cancer screening.

**Results:** Among 236 participants, most were Asian (74%), non-US born (79%), and privately insured (57%). One-third responded in Chinese (37%). Half reported having ≥1 HRSN. Interpersonal violence was high across all participants. Transportation needs were highest among Chinese-T participants, while food insecurity and housing instability were higher among Chinese-S participants. Lower-income patients had higher odds of having ≥2 HRSN (OR:2.53, 95% CI: 1.12, 5.98). Older age and public insurance/uninsured were significantly associated with low neighborhood perceptions.

**Conclusions:** We observed higher than anticipated reports of HRSN among primary care patients in a suburban, hospital-affiliated practice serving AA. Low neighborhood perceptions, particularly among Chinese-S participants, highlight the importance of addressing broader SDOH among insured, suburban AA patients. These study findings inform the need to augment HRSN identification to adequately address social needs that impact health outcomes and life course experiences for Asian patients. As HRSN measuring efforts continue, and COVID-19's impact on the health of minority communities emerge, it will be critical to develop community-specific referral pathways to connect AA to resources for HRSN and continue to address more upstream social determinants of health for those who are disproportionately impacted.

## Introduction

The recognition that social determinants of health and structural barriers drive inequities in health and heath care has long been a central tenet in public health (1–6). However, there is recent focus to address social determinants of health within health care settings (7–17) as a way to reduce higher rates of chronic disease and poorer outcomes among vulnerable patients (18–20). Health related social needs (HRSN), including transportation, food insecurity, housing stability and interpersonal violence which are more downstream factors that impact health care, also result directly in both poorer outcomes and weaker health system performance (17, 21–23). Thus, efforts to systematically collect information on HRSN in clinical settings and develop solutions to address HRSN are on the rise (12, 13, 16, 23–32). Few clinic-based HRSN studies to date, however, have focused on measuring HRSN in languages other than English or Spanish or in diverse immigrant populations, including Asian Americans (AA), where cultural factors and immigration experiences can pose additional barriers to accessing care and routine preventive services (33). Furthermore, AA patients as a group are often masked by small or aggregated numbers and a lack of linguistically appropriate measurement tools within larger health system settings (34–36).

AAs are among the fastest growing populations in the United States (US) and New Jersey ranks third among states having the largest proportion of AA residents (>10%), following California and Hawaii (37, 38). Chinese Americans are the largest AA subgroup and nearly two-thirds of Chinese-Americans are born outside of the US (39). Within New Jersey, Chinese Americans are the second largest AA ethnic group and the majority reside in Northern and Central New Jersey counties (38). Prior data indicate specific AA populations experience higher rates of chronic disease and poorer mental well-being compared to non-minorities (40–44). Many—particularly the elderly – may experience significant emotional or psychosocial distress, lower levels of social engagement (45), and low health literacy (46). Additional socioeconomic and cultural barriers unique to Asian immigrants further contribute to disparities in access to health care, uptake of preventive screenings, and adherence to chronic disease management for AA populations (45, 47, 48). Factors related to trauma and immigration experiences, as well as resilience that is developed through the coping of these life events, can negatively and positively impact their health outcomes (16, 49–53). Perceptions of neighborhood, including social cohesion and trust can also impact health care utilization and outcomes (54, 55). Thus, focusing on improving the measurement of HRSN within clinic settings for AA populations, and providing in-language screening tools for larger population groups, such as Chinese patients, can inform broader health system strategies to address population level unmet social needs.

Cancer inequities among AAs are a prime example of the influence of HRSN on health disparities. Cancer is the first leading cause of death in the US for AAs and the second leading cause of death among other racial/ethnic groups. Breast cancer mortality rates in immigrant AA women are higher compared to US-born counterparts (56). Socioeconomic factors, income, and transportation-related barriers have all been implicated in cancer screening disparities among AA immigrants. Lower rates of cancer screening have been observed in AAs (57), but also specifically in Chinese Americans (46). For example, Chinese Americans have some of the lowest rates of breast and cervical cancer screening among all AA subgroups (58). In New Jersey, rates of colorectal cancer screening were lowest among Asians in 2012–2016 compared to all other racial/ethnic groups (59). Unless targeted efforts are made to develop appropriate HRSN screening tools for AAs within clinic settings, newly implemented tools to address population health and health care disparities, including for cancer, will be limited for AA populations.

This study aims to understand and more accurately assess the prevalence of HSRN and neighborhood perceptions among AA primary care patients, using an adapted HRSN screening tool among patients in a suburban primary care practice in New Jersey. At study initiation, which occurred ~6 months prior to the COVID-19 pandemic, we hypothesized that a higher proportion of lower-income and more recently immigrated patients would report having HRSN and lower neighborhood perceptions. We examined the relationship between HRSN and neighborhood perceptions on history of prior breast or colorectal cancer screening among age-eligible study participants as an exploratory assessment of the impact of HRSN on preventive care utilization. Given that our study period intersected with the COVID-19 pandemic and associated discrimination against AA communities, we further compared reports of HRSN and neighborhood perceptions between participants recruited before and during the pandemic.

## Materials and Methods

### Study Setting and Target Population

We assessed HRSN, neighborhood perceptions (social cohesion, trust), and immigration characteristics (time since immigration, birthplace) through a cross-sectional survey among established patients at the Center for Asian Health (CAH), an outpatient primary care practice of Saint Barnabas Medical Center, a community hospital in suburban New Jersey belonging to the RWJBarnabas Health system. The Center for Asian Health was started in 2013 with the goal of meeting the healthcare needs of the growing Chinese American population in Northern New Jersey. CAH sees 5,000 patient visits per year with a mix of primary care providers and specialists. In March 2020 when COVID-19 stay-at-home orders began, patients were exclusively seen via telehealth until restrictions eased in June 2020. In-person office visits increased by late July but then were scaled back in Fall 2020 when community COVID-19 transmissions increased again regionally.

### Recruitment

This study was approved by the Rutgers Biomedical Health Sciences Institutional Review Board and the Saint Barnabas Medical Center Institutional Review Board. Adult patients age 18 and over who could complete the survey in English or Chinese were approached to participate in the study. New patients and those who could not complete a survey in English or Chinese were excluded. Recruitment occurred between September 2019 and November 2020. Prior to the COVID-19 pandemic (September 2019-March 2020), research team members, including bilingual Mandarin/Cantonese speaking CAH clinic volunteer staff, approached patients in the waiting room to introduce the study components and assess interest in participation. Interested patients were then screened for study eligibility and asked to review and sign a written informed consent form.

Following COVID-19 stay-at-home orders (May 2020-August 2020), CAH patients with telehealth visits were invited to participate in the study through the CAH patient portal or via email. If they agreed, the eligibility screener, consent, and survey were then completed online via REDCap. When in-person primary care visits resumed more broadly during the COVID-19 pandemic (September 2020-November 2020), recruitment via in-person visits was reinitiated, with an additional option of completing the eligibility screener, consent form, and survey at home, either online or via paper/pencil to be mailed back to the clinic.

### Survey Administration

The 38-item survey instrument, which took participants ~10–15 min to complete, was available electronically on iPads in English and Chinese [Chinese-Traditional (T), Chinese-Simplified (S)], for study participants to complete in the waiting room or in the clinic exam room following the in-person enrollment procedures described above. Both Chinese-T and Chinese-S survey language text were made available based on clinic staff and provider input about language needs of the CAH patient population. Paper surveys were available for in-person participants upon request. During the COVID-19 pandemic, participants were provided a survey link via email or the patient portal to complete online at home. Participants who completed the survey received a $10 gift card which was given either in person (if completed in person) or mailed to them (if completed online or via mail).

### Survey Measures

The 38-item survey instrument included: health-related social needs screening items, a neighborhood perception scale, and measures used in prior studies to assess immigration experiences, trauma, and sociodemographic factors (60).

#### Health Related Social Needs

Our HRSN measures were based on the 2016 Centers for Medicare and Medicaid Services (CMS) screening tool for HRSN through CMS Accountable Health Communities (61), which comprised of a ten-item (27, 36, 62–65) HRSN Screening Tool covering the following social needs: housing instability (“*What is your living situation today?”*), food insecurity (2 questions: “*Within the past 12 months, you worried that your food would run out before you got money to buy more;” “Within the past 12 months, the food you bought just didn't last and you didn't have money to get more.”*), transportation (“*In the past 12 months, has lack of reliable transportation kept you from medical appointments, meetings, work or from getting to things needed for daily living?”*), utility needs (“*In the past 12 months has the electric, gas, oil, or water company threatened to shut off services in your home?”*), and interpersonal violence (4 questions: “*How often does anyone, including family and friends, physically hurt you?” “How often does anyone, including family and friends, insult or talk down to you?” “How often does anyone, including family and friends, threaten you with harm?” “How often does anyone, including family and friends, scream or curse at you?”*)

We created composite measures for each domain that had more than one question with yes (at least one reported need within the domain) and no (answer no to all questions in the domain). We also created an HRSN composite measure by aggregating the number of unmet HRSN reported and then categorized overall HRSN as none vs. 1 or more, and none/one vs. 2 or more. We compared frequency distributions of HRSN reported by study participants with frequencies reported from the 2019 New Jersey statewide Health and Well-Being Poll (66). The Health and Well-Being Poll was developed by the Rutgers Center for State Health Policy with funding from the Robert Wood Johnson foundation. Abt Associates, under contract to Rutgers, drew a statewide random digit dialed telephone (landline and cell) sample and conducted interviews in English and Spanish, from January to February 2019 with adults living in New Jersey.

#### Neighborhood Perceptions

We measured neighborhood perceptions related to connectedness, belonging, and trust using a 12-item scale, previously implemented in other studies (67–70), including studies focused on elderly Chinese Americans (71). For each item, participants were asked to respond to a five-point scale (strongly disagree, disagree, neutral, agree or strongly agree). A composite score was constructed using the sum of all 12 items and then dichotomized to low (unfavorable) neighborhood perceptions (total score ≤ 36) and high (favorable) neighborhood perceptions (total score >36). Participants with missing data for any single item were still included in the final analysis, and were included within the “disagree” category for those items.

#### Other Sociodemographic Factors

We examined the following sociodemographic variables from the survey: gender, age (18–49, 50–65, >65 years), survey language (English, Chinese-Traditional (T), Chinese-Simplified (S)), race/ethnicity (Non-Hispanic-Asian, Other race/ethnicity), household income (< $75,000; $75, 000 or more; unknown/missing), education level (less than college, college or beyond), and insurance status (private insurance, Medicaid/Medicare, uninsured/unknown). Participants reported whether they were born in the US or born outside of the US, as well as indicating country of birth. We calculated percent of life spent in the US using current age and age at time of immigration among those born outside of the US and constructed the following mutually exclusive categories: US-born, <25% of life spent in US, ≥25% of life spent in the US.

### Medical Record Chart Abstraction

We abstracted data from CAH's electronic medical records (Cerner PowerChart) for patients age-eligible (51–75 years) for routine breast (females only) and colorectal (females and males) cancer screening based on current US Preventive Services Task Force (USPSTF) guidelines (72, 73). Records were reviewed retrospectively until the timing of the last screening service was able to be identified or up until 10 years from the survey date. For patients who did not have a complete 10-year look-back period, we reviewed all records that were retrospectively available.

#### Cancer Screening

We examined ever-screened vs. never-screened and receipt of guideline-concordant screening (yes/no) for both breast and colorectal cancer among eligible survey participants. Receipt of guideline-concordant breast cancer screening was determined as whether a mammogram was received within the last 2 years from the time of survey completion. We excluded women who had undergone bilateral mastectomies (*n* = 2). Receipt of guideline-concordant colorectal cancer screening was determined as either receiving a colonoscopy within the last 10 years, or completing a multi-target stool DNA test within the last 12 months (72). We recorded whether screening was ever received, as well as the year they were last received. Regardless of whether the participants completed the survey in 2019 or 2020, we used 2019 as the year to start the look-back period for retrospective chart review for all participants to employ a more inclusive approach of whether screening occurred within guideline recommendations.

### Statistical Analysis

Descriptive statistics were used to summarize the demographic characteristics, HRSN variables, neighborhood perception, and enrollment pre-COVID-19 vs. during COVID-19 among eligible study participants age 18 and over in the final analytic sample, and compared across survey language using Chi-square test and Fisher's exact test. We used survey language (English, Chinese-T, Chinese-S) as a primary comparison based on observed demographic differences across the Chinese language groups in our study sample as well as input and feedback from CAH clinic staff and community partners from the Asian Health Initiative at the Rutgers Institute for Health, Healthcare Policy & Aging Research.

**Figure 1:**
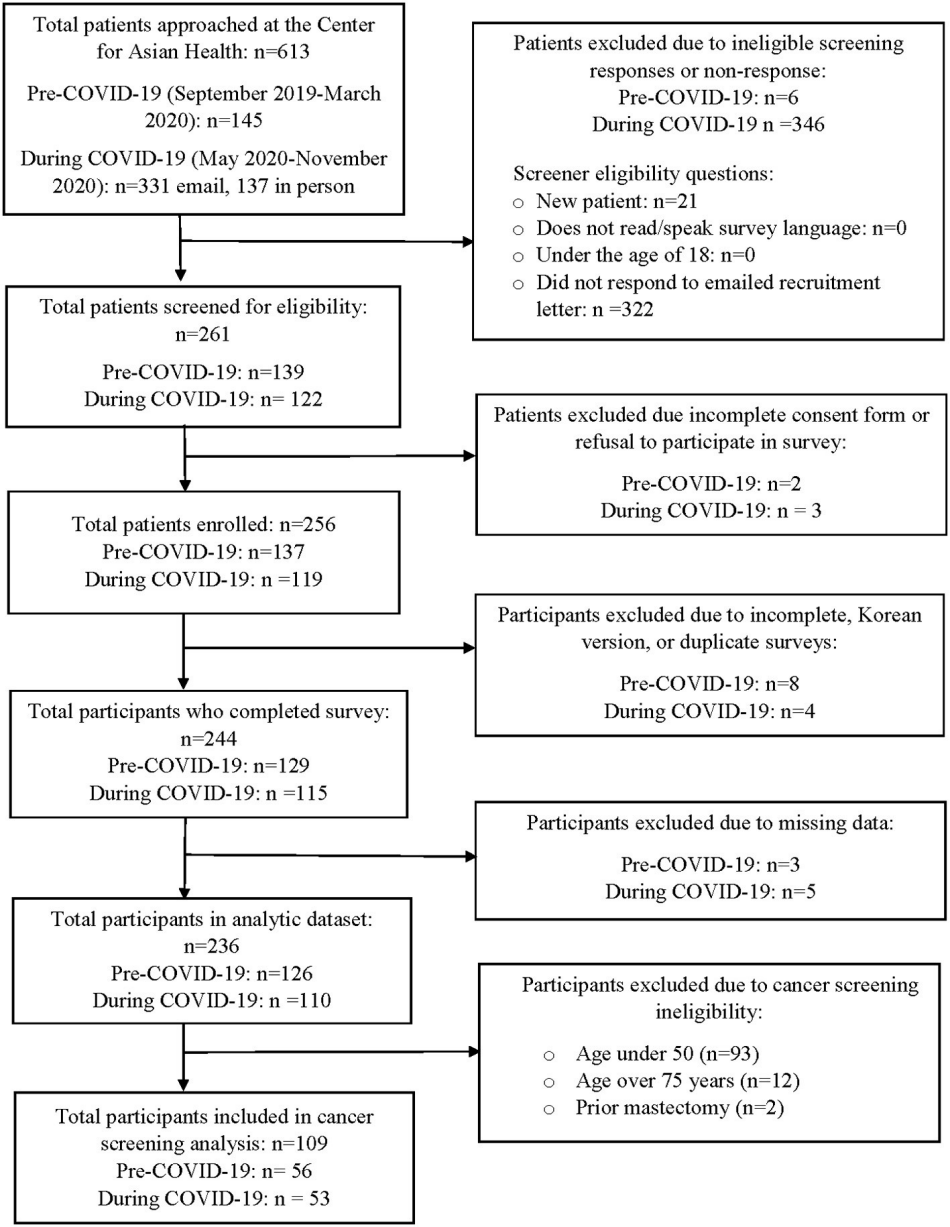
Recruitment and study enrollment (September 2019 to November 2020).

A total of 12 participants were excluded due to incomplete or duplicate responses or surveys completed in Korean (*n* = 1). While a Korean survey instrument was available at the start of our study, study recruitment for Korean participants was limited due to staff availability and appointments with the Korean speaking provider were only available 1 day a week. For surveys that were missing information on age (*n* = 24), we used the medical record to determine the missing ages of these participants. We were able to identify missing ages for 23 participants. An additional 8 participants were excluded due to missing data for other variables of interest. The final analytic sample for the primary analysis included 236 participants (Figure 1). In univariate analyses we compared reports of having ≥2 HRSN compared to reporting ≤ 1 HRSN, as well as low neighborhood perceptions (score <36) compared to high neighborhood perceptions (score ≥ 36) using logistic regression models. We ran separate multivariable models to determine sociodemographic factors associated with reporting ≥2 HRSN and low neighborhood perceptions. Independent variables in the final models were based on significant univariate associations, prior literature, and the overall analytic sample size. We report odds ratios (OR) and 95% confidence intervals (CIs).

We also compared receipt of cancer screening (ever vs. never; guideline-concordant vs. non-guideline-concordant) by report of HRSN and neighborhood perceptions using Chi-square test and Fisher's exact test among study participants who completed the survey and age-eligible for cancer screening. Participants were excluded from the analysis who were not age-eligible for screening at the time of survey, unable to be matched in the medical record based on the recorded survey name, or had no name on the survey (Figure 1). Only descriptive analyses were examined for cancer screening because the primary study sample was not powered to examine the association between HRSN or neighborhood perceptions and receipt of screening, as well as the limited frequency distribution in cancer screening measures within the study population. All analyses were conducted in R version 4.0.3 (74).

## Results

Characteristics of the study participants (*n* = 236) are shown in Table 1. The majority of participants were recruited in-person and completed the survey via tablet with the exception of 21 participants. A large proportion of participants self-identified as being a woman (64%) and non-Hispanic Asian (75%), having a college degree (68%), and having an annual income of more than $75,000 (52%). The mean age at the time of the survey was 52.6 years.

**Table 1:** Characteristics of study participants by survey language.

	**Total** ***N*** ***=*** **236**		**English** ***N*** **=** **149**		**Chinese traditional** ***N*** **=** **43**		**Chinese simplified** ***N*** **=** **44**		
**Characteristic**	**n**	**%**	**n**	**%**	**n**	**%**	**n**	**%**	***p*-value**^**a**^
Race/ethnicity									<0.001
NH-Asian	175	74.2	94	63.0	41	95.0	40	91.0	
Other race/ethnicity	61	25.8	55	37.0	2	4.7	4	9.1	
Gender									0.19
Female	154	65.3	103	69.0	27	63.0	24	55.0	
Male	82	34.7	46	31.0	16	37.0	20	45.0	
Age									0.112
18–49	93	39.4	65	44.0	11	26.0	17	39.0	
50–65	92	39.0	58	39.0	17	40.0	17	39.0	
>65	51	21.6	26	17.0	15	35.0	10	23.0	
Education									0.022
Less than college	70	30.0	38	26.0	12	28.0	20	48.0	
College or beyond	163	70.0	110	74.0	31	72.0	22	52.0	
Health insurance									0.008
Private	135	57.2	98	66.0	21	49.0	16	36.0	
Medicaid/Medicare	73	30.9	36	24.0	16	37.0	21	48.0	
Uninsured/unknown	28	11.9	15	10.0	6	14.0	7	16.0	
Income									0.044
Less than $75K	95	40.3	61	41.0	11	26.0	23	52.0	
$75K or more	122	51.7	79	53.0	27	63.0	16	36.0	
Unknown/missing	19	8.1	9	6.0	5	12.0	5	11.0	
Percent life spent in US									<0.001
US-born	49	20.8	49	37.0	0	0.0	0	0.0	
<25 in the US	23	9.7	6	4.5	5	12.0	12	34.0	
25–99 in the US	138	58.5	79	59.0	36	88.0	23	66.0	
Health-related social needs									0.533
None	117	49.6	78	52.0	19	44.0	20	45.0	
At least 1	119	50.4	71	48.0	24	56.0	24	55.0	
Health-related social needs									0.078
1 or fewer	202	85.6	132	89.0	37	86.0	33	75.0	
At least 2	34	14.4	17	11.0	6	14.0	11	25.0	
Neighborhood perceptions score									0.002
Low (36 or lower)	47	19.9	25	17.0	5	12.0	17	39.0	
High (>36)	189	80.1	124	83.0	38	88.0	27	61.0	
Recruitment period									0.133
Pre-COVID-19	126	53.4	73	49.0	24	56.0	29	66.0	
During COVID-19	110	46.6	76	51.0	19	44.0	15	34.0	

a *Statistical tests performed: chi-square test of independence; Fisher's exact test*.

Over three quarters of participants were born outside of the U.S. (*n* = 187, 79%). All participants who completed the survey in Chinese-T (19%) and Chinese-S (18%) were non-US born, with the most common location of birth being China (*n* = 70) or Taiwan (*n* = 45). There were observed differences in demographic characteristics across the three survey language groups and in immigration characteristics between Chinese-T and Chinese-S survey participants. Compared to Chinese-T respondents, higher proportions of Chinese-S respondents reported having incomes less than $75K (52% vs. 26%), residing in the US for <25% of their lifetime (34% vs. 12%), and having less than a college degree (48% vs. 28%). Overall, a lower proportion of Chinese-S respondents were recruited during the COVID-19 pandemic period (34%) compared to Chinese traditional (44%) and English (51%) respondents.

### Health Related Social Needs

Half of all participants (50%) reported having at least one HRSN, with minimal differences across language groups (English: 48%, Chinese-T: 56%, Chinese-S: 55%; *p*-value: 0.533). While a smaller proportion of overall participants reported having ≥2 HRSN (14%), larger differences were observed across survey language. Compared to 14% of Chinese-T and 11% of English respondents, a quarter of Chinese-S respondents reported having ≥2 HRSN (Table 1).

**Figure 2:**
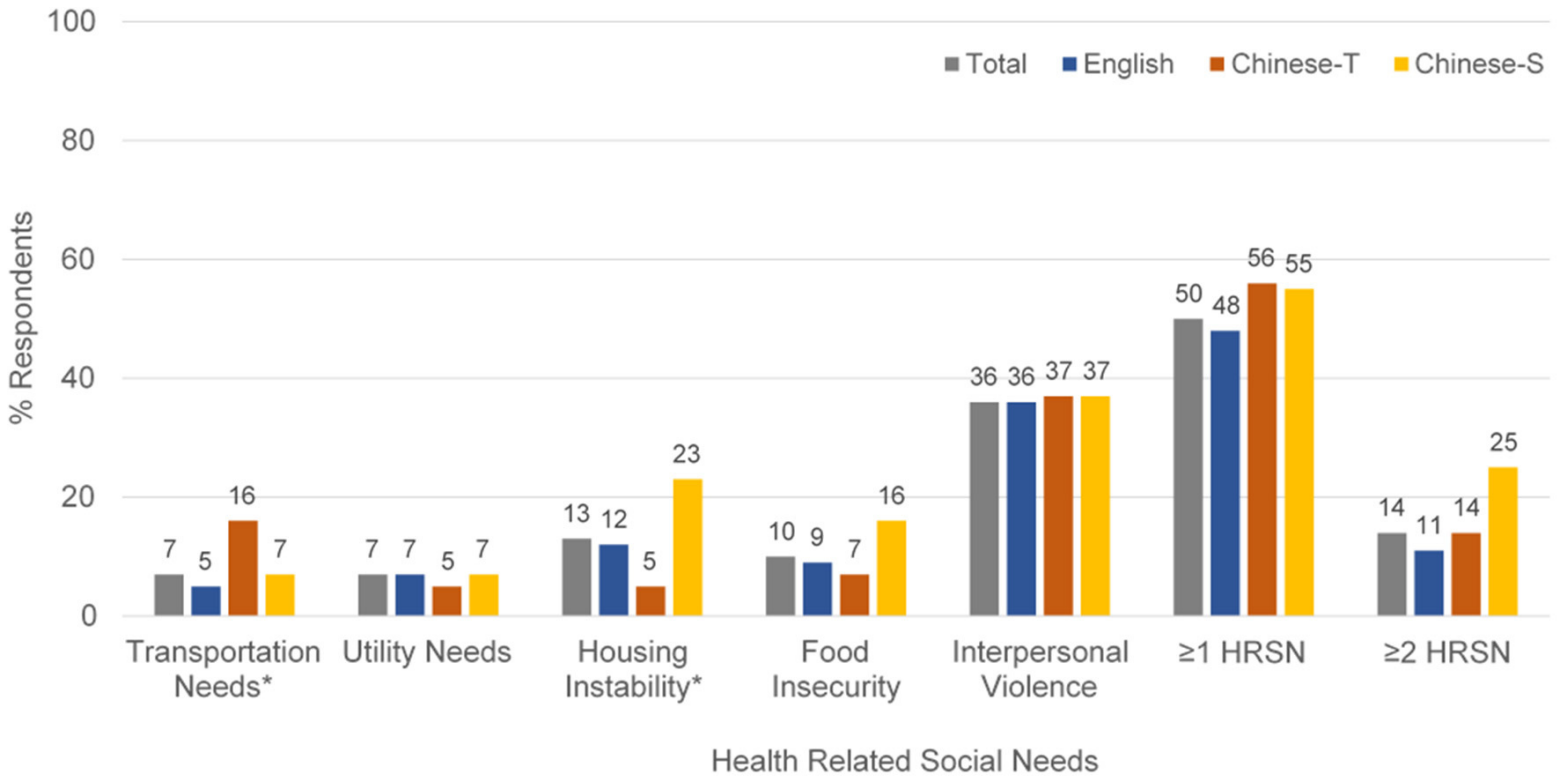
Health related social needs by survey language among all study participants (*n* = 236). *p < 0.05.

Higher proportions of Chinese-S respondents reported housing instability (23%) compared to both English (12%) and Chinese-T respondents (5%) (*p*-value: 0.038) (Figure 2). Similarly, though not statistically significant, higher proportions of Chinese-S respondents reported food insecurity (16%) compared to both English (9%) and Chinese-T respondents (7%) (*p*-value: 0.317) (Figure 2). While Chinese-T respondents were the least likely to report experiencing utility needs (5%), they were more likely to report having transportation needs (16%) than either English (5%) or Chinese-S (7%) respondents (*p*-value: 0.039). More than one-third of all respondents reported experiencing at least one interpersonal violence measure across all three survey languages.

When comparing these findings to statewide data from the New Jersey Health & Well-Being Poll (Table 2), transportation needs were higher in our sample of Chinese-T participants (16%) compared to New Jersey residents overall (6%). Food insecurity was also higher among our sample of Chinese-S participants (23%) compared to New Jersey residents (11%).

**Table 2:** Comparison of study participants reporting HRSN to state data.

	**% Transportation needs**	**% Utility needs**	% Housing instability^*^	**% Food insecurity**	% Interpersonal violence^**^
**Study participants**				
English	4.7	7.4	3.3	9.4	26.4
Chinese-T	16.0	4.8	0.0	7.0	34.9
Chinese-S	6.8	6.8	6.8	16.0	27.9
New Jersey Health & Well-Being Poll	6.5	5.9	6.9	11.2	13.0

* *Housing instability based on comparable survey item only (current living situation), not composite measure shown in Figure 1*.

** *Interpersonal violence measure also based on comparable survey item only (screamed or cursed at by loved one), not composite measure shown in Figure 1*.

In the univariate analysis, Chinese-S participants (OR: 2.59; 95% CI: 1.08, 6.01) had higher odds of having ≥2 HRSNs compared to English survey respondents (Table 3). Similarly, participants with lower income (< $75,000) had higher odds (OR: 2.52, 95% CI: 1.15, 5.77) of having ≥2 HRSNs compared to higher income participants. After adjusting for gender, age, and recruitment period, participants with incomes < $75,000 still had higher odds of reporting ≥2 HRSNs (OR: 2.53; 95% CI: 1.12, 5.98) compared to higher income participants.

**Table 3:** Factors associated with reporting ≥ 2 HRSN and low neighborhood cohesion (*n* = 236).

	**≥2 HRSN**				**Low neighborhood perceptions**			
	**Univariate models**		**Multivariable model**		**Univariate models**		**Multivariable model**	
	**OR**^**a**^	**95% CI**^***a***^	**OR**	**95% CI**	**OR**	**95% CI**	**OR**	**95% CI**
**Gender**							
Male	Ref	Ref	Ref	Ref	Ref	Ref	Ref	Ref
Female	0.97	0.46, 2.14	1.00	0.45, 2.28	1.50	0.76, 3.13	1.74	0.81, 3.94
**Age**							
18–49	Ref	Ref	Ref	Ref	Ref	Ref	Ref	Ref
50–65	0.61	0.26, 1.36	0.56	0.23, 1.30	1.01	0.49, 2.08	0.97	0.43, 2.17
>65	0.60	0.20, 1.55	0.46	0.15, 1.28	0.83	0.33, 1.97	**0.34**	**0.11, 0.98**
**Survey language**							
English	Ref	Ref	Ref	Ref	Ref	Ref	Ref	Ref
Chinese simplified	**2.59**	**1.08, 6.01**	2.40	0.96, 5.87	**3.12**	**1.48, 6.58**	2.26	0.95, 5.29
Chinese traditional	1.26	0.43, 3.27	1.66	0.54, 4.64	0.65	0.21, 1.70	0.59	0.17, 1.70
**Income**							
$75K or more	Ref	Ref	Ref	Ref	Ref	Ref	Ref	Ref
Less than $75K	**2.52**	**1.15, 5.77**	**2.53**	**1.12, 5.98**	**3.33**	**1.64, 7.08**	1.93	0.85, 4.47
Unknown/missing	2.69	0.68, 9.06	2.68	0.65, 9.42	**4.89**	**1.59, 14.6**	**3.78**	**1.12, 12.2**
**Health insurance**							
Private	Ref	Ref			Ref	Ref	Ref	Ref
Medicaid/Medicare	2.07	0.94, 4.55			**3.42**	**1.68, 7.13**	**3.87**	**1.50, 10.3**
Uninsured/unknown	1.33	0.36, 4.06			**2.97**	**1.09, 7.75**	**3.01**	**1.00, 8.77**
**Recruitment period**							
Pre-COVID-19	Ref	Ref	Ref	Ref	Ref	Ref	Ref	Ref
During COVID-19	0.77	0.36, 1.60	0.89	0.41, 1.93	0.81	0.42, 1.55	0.93	0.44, 1.92

a *OR, Odds Ratio; CI, Confidence Interval. Bolded items represent significant associations at the p < 0.05 level*.

### Neighborhood Perceptions

**Figure 3:**
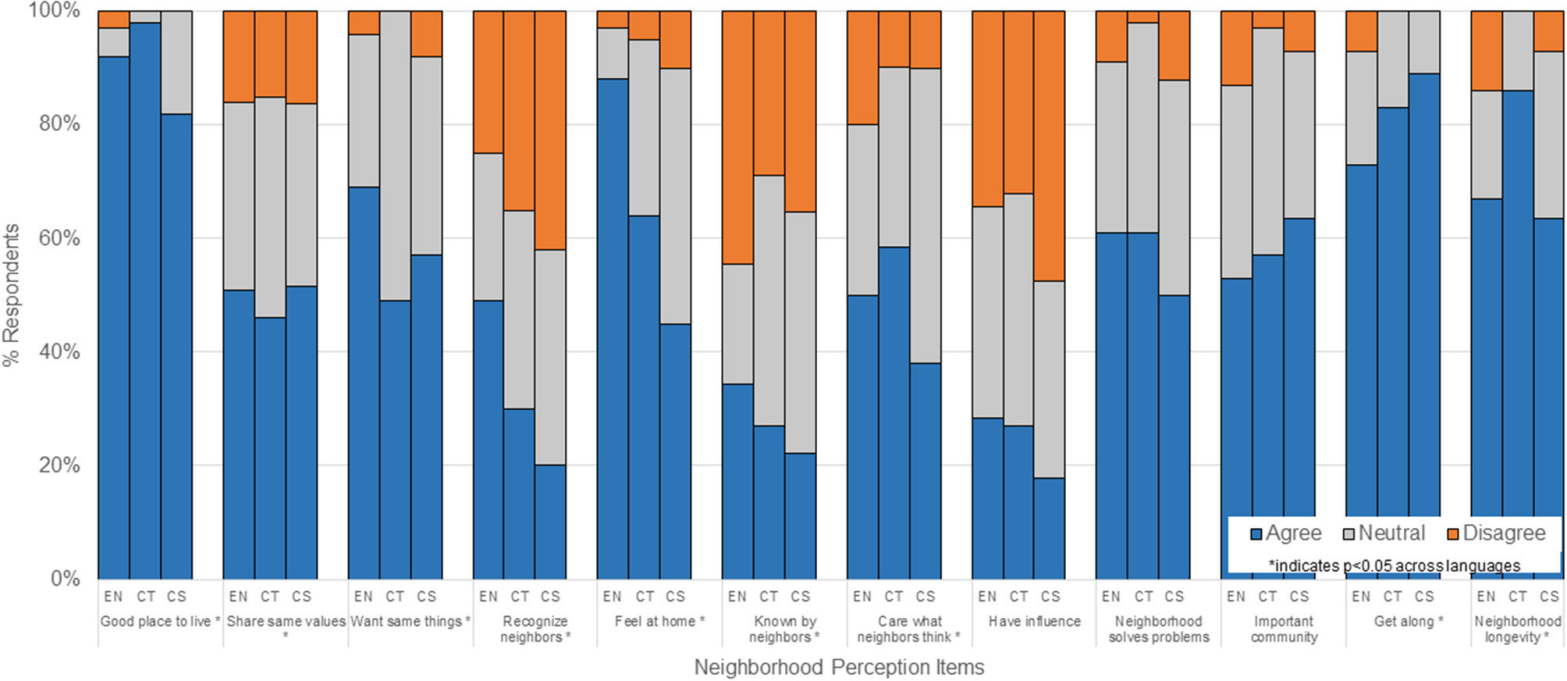
Neighborhood perceptions among study participants by survey language.

Neighborhood perceptions among study participants are shown in Figure 3. Significant differences in neighborhood perceptions were observed across survey language. A larger proportion of Chinese-S survey respondents (39%) had low neighborhood perceptions (score ≤ 36) compared to 12% Chinese-T respondents and 17% English respondents (*p* = 0.002). For individual neighborhood perception items, no Chinese (simplified or traditional) respondents disagreed with the fact that their neighborhood was a good place to live, although nearly one-fifth of Chinese-S respondents felt “neutral” (*p* = 0.029). More Chinese-S (42%) and Chinese-T (35%) respondents did not feel they could recognize their neighbors compared to English respondents (25%), suggesting differences in neighborhood belonging and familiarity. Lower proportions of Chinese survey respondents agree that they feel at home in their neighborhood (Chinese-S 45%, Chinese-T 64%, English 89%; *p* < 0.001). Chinese-T respondents (86%) were much more likely to expect to live in their neighborhood for a long time than either English (67%) or Chinese-S (63%) respondents.

In the univariate analysis (Table 3), Chinese-S respondents, participants with lower incomes, and patients with Medicaid/Medicare had significantly higher odds for reporting low overall neighborhood perceptions. In the adjusted model including participants in all languages and after adjusting for gender, age, health insurance, and recruitment period, older participants (age > 65 years) had lower odds of having low neighborhood perceptions (OR: 0.34; 95% CI: 0.11, 0.98) compared to younger participants, whereas those with Medicaid/Medicare (OR: 3.87; 95% CI: 1.50, 10.3) had higher odds of having low neighborhood perceptions compared to privately insured participants. A similar relationship was observed for participants who were uninsured or had unknown insurance compared to privately insured participants (OR: 3.01; 95% CI: 1.00, 8.77). In our sensitivity analysis of participants who were missing responses for 6 or more of the 12 neighborhood perception items (*n* = 6), associations between uninsured/unknown insurance and low neighborhood perceptions and low income/unknown income and low neighborhood perceptions were no longer significant (data not shown).

### Relationship Between HRSN & Low Neighborhood Perception on Cancer Screening

Our exploratory analysis of cancer screening history and health-related social needs included 67 women age-eligible for breast cancer (BC) screening and 109 men and women eligible for colorectal cancer (CRC) screening. Ever receiving a prior BC screening (87%) or CRC screening (81%) as well as having a guideline concordant screening history (BC: 72%, CRC: 76%) were high among patients who participated in our HRSN screening assessment (Table 4). We did not observe significant differences in ever receiving a previous BC or CRC screening, or receipt of guideline-concordant BC or CRC screening, by HRSN status or neighborhood perceptions. For BC screening, although not statistically significant, we observed a slightly higher proportion of those with fewer (≤ 1) HRSN who did not receive guideline-concordant screening (29%), compared to those with ≥2 HRSN (22%). A similar percentage of patients reporting ≥2 HRSN did not receive guideline-concordant CRC screening (23%), compared to those with ≤ 1 HRSN (24%). For BC screening, a similar percentage of patients reporting low neighborhood cohesion had never been screened (13%), compared to those reporting high neighborhood cohesion (13%).

**Table 4:** Breast or colorectal cancer screening history among age-eligible participants by HRSN and neighborhood perceptions.

	**Breast Cancer (*****n*** **=** **58)**				**Colorectal Cancer (*****n*** **=** **109)**			
	**Ever screened**	**Never screened**	**Guideline concordant**	**Non-guideline concordant**	**Ever screened**	**Never screened**	**Guideline concordant**	**Non-guideline concordant**
Total	86.6%	13.4%	71.6%	28.4%	80.7%	19.3%	76.1%	23.9%
**HRSN**								
None	80.0%	20.0%	62.9%	37.1%	82.1%	17.9%	76.8%	23.2%
1 or more	93.8%	6.0%	81.3%	18.8%	79.2%	20.8%	75.5%	24.5%
**HRSN**								
None or 1	86.2%	14.0%	70.7%	29.3%	80.2%	19.8%	76.0%	24.0%
2 or more	88.9%	11.0%	77.8%	22.2%	84.6%	15.4%	76.9%	23.1%
**Transportation needs**								
None	85.0%	15.0%	70.0%	30.0%	81.0%	19.0%	77.0%	23.0%
Yes	100.0%	0.0%	85.7%	14.3%	77.8%	22.2%	66.7%	33.3%
**Utility needs**								
None	86.2%	14.0%	70.8%	29.2%	81.7%	18.3%	76.9%	23.1%
Yes	100.0%	0.0%	100.0%	0.0%	60.0%	40.0%	60.0%	40.0%
**Living situation needs**								
None	86.2%	14.0%	70.7%	29.3%	81.4%	18.6%	78.4%	21.6%
Yes	88.9%	11.0%	77.8%	22.2%	75.0%	25.0%	58.3%	41.7%
**Food insecurity**								
None	88.5%	11.0%	72.1%	27.9%	79.8%	20.2%	75.8%	24.2%
Yes	66.7%	33.0%	66.7%	33.3%	90.0%	10.0%	80.0%	20.0%
**Interpersonal violence**								
None	80.0%	20.0%	66.7%	33.3%	80.6%	19.4%	75.0%	25.0%
Yes	100.0%	0.0%	85.0%	15.0%	82.9%	17.1%	80.0%	20.0%
**Neighborhood perception**								
Low (36 or lower)	86.7%	13.3%	73.3%	26.7%	70.0%	30.0%	65.0%	35.0%
High (>36)	86.5%	13.5%	71.2%	28.9%	83.1%	16.9%	78.7%	21.4%
**Recruitment period**								
Pre-COVID-19	88.6%	11.0%	77.1%	22.9%	82.1%	17.9%	76.8%	23.2%
During COVID-19	84.0%	16.0%	65.6%	34.4%	79.2%	20.8%	75.5%	24.5%

## Discussion

This is one of the few clinic-based studies to implement a HRSN screening tool in languages other than English and Spanish and assess the prevalence of social needs among patients in an Asian American focused primary care clinic. We found similar, and in some cases higher, reports of health-related social needs within our study population of primarily privately-insured, higher educated, suburban non-Hispanic Asian patients compared to statewide New Jersey data.

We observed alarmingly higher than anticipated reports of interpersonal violence across all survey languages. While these reports did not significantly increase among participants recruited during the COVID-19 pandemic compared to those recruited prior to the start of the COVID-19 pandemic, we observed higher rates of interpersonal violence needs across participants in both time periods and all three survey languages when compared to similar measures in the state level data. These findings warrant focused efforts for developing clinic strategies in increasing clinician awareness, follow-up and referral processes, and longer-term interventions to address interpersonal violence as a health-related social need among AA primary care patients. These data also substantiate the need to address the “Invisible Minority” status of Asian Americans, as interpersonal violence risks are just as prevalent in our sample as state reported rates. The wave of recent hate crimes directed toward Asian Americans during the COVID-19 pandemic (75), unfortunately indicate interpersonal violence is increasing. A March 2021 Pew survey found that nearly three-quarters of people (71%) feel that AAs experience “a lot” or “some” discrimination (76). Other reports have cited up to a tenfold increase in the number of reports of anti-Asian sentiment, including verbal harassment and physical assault in the larger New York City region in February-March 2021 compared to the same period in 2020 (76). The anti-Asian hate and violence will not only increase the mental health care needs of AAs going forward, but also impact long-term access, utilization and adherence to health services overall, leading to downstream effects of increased chronic conditions (77).

Housing instability and food insecurity needs were also notably high among participants who completed the survey in simplified Chinese text, which comprise of participants who are younger and immigrated to the US in more recent years. Although food insecurity has decreased since 2011, the COVID-19 pandemic has caused rates to double (78). Similarly, homelessness has decreased overall since 2007, but has increased annually between 2017 and 2019 (79). Efforts to address social needs within health care settings as a strategy to improve overall health among AA specifically has been limited, despite the emphasis on measuring and addressing HRSN among low-income and other racial/ethnic minority patients (12, 27, 80). Our findings indicate vulnerability to HRSN among patients in a clinic setting serving AA who are higher-income and privately insured. These findings are consistent with other work that challenge the Model Minority Myth (45, 60, 81) for AA and further support the need to adapt and tailor existing clinic-based HRSN assessment and intervention/referral strategies to address the social needs and life course experiences of heterogeneous AA populations (82). At a minimum, broader efforts to screen HRSN within clinic populations need to be linguistically appropriate for AA patients.

Our findings also point to higher transportation needs among Chinese American patients who completed the survey in traditional Chinese, which consist of participants who are older overall compared to English and simplified Chinese survey participants. Access to transportation has been identified in prior studies among elderly AA and other racial/ethnic minority populations as logistical barriers to accessing health care (8, 48). For elderly AA patients in suburban areas, such as New Jersey, where public transportation options are limited and social services support programs for those who have limited English proficiency are more disperse, overcoming transportation needs to health care may be a greater obstacle compared to AA patients in more urban centers. The majority of CAH patients reside across three counties in Northern New Jersey, however, a number of patients live outside of these immediate areas and seek care from CAH, often citing the in-language care as the reason for traveling further. A recent report on the State of AA in New Jersey, indicated that Chinese Americans are the second largest Asian American ethnic group in the state following Indian Americans, with a large proportion residing in Northern and Central New Jersey counties (38). In-language healthcare is often not geographically close for residents of suburbs and access becomes an issue, especially for those who cannot drive. It is important to monitor and address these needs for suburban AA communities living outside of densely populated ethnic enclaves, such Chinatowns in Manhattan and Brooklyn, as they can contribute to health disparities and poorer outcomes. Linguistic and geographic challenges to health care are often not detected in aggregated data and similar to the bimodal distribution of income in AA, which is often masked in population data within suburban areas (55).

In our assessment of neighborhood perceptions, we also observed notable differences across survey language groups. Chinese participants responding in simplified text reported more negative perceptions about their neighborhood compared to English and traditional Chinese text respondents, including fewer Chinese-S participants “feeling at home” or “knowing their neighbors.” We did observe slight increases in specific measures between the pre-COVID-19 vs. during COVID-19 periods, including “feeling at home” (from 49% to 51%) and “caring about what neighbors think” (from 46% to 54%). Similar to our findings for interpersonal violence described above, we did not observe significant differences in overall low neighborhood perceptions between participants recruited during the COVID-19 pandemic compared to those recruited before the COVID-19 pandemic began. We may not have observed significant differences in HRSN, specifically interpersonal violence, or neighborhood perceptions between COVID-19 periods because of a bias in the patients who were seeking and able to receive health care during the COVID-19 pandemic period. Patients who make it to a primary care encounter either in-person or through telehealth, may be less likely to have lost health insurance, be less vulnerable to having HRSN, less likely to have experienced fear or trauma from COVID-19 and related social impacts, or more chronically ill and requiring primary care follow-up. Prior research on neighborhood cohesion have highlighted how AA communities may mitigate disparities and cultural stress for AA patients, showcasing the potential protective effects of community interactions (19). On the other hand, AA living in more suburban and less densely AA populated areas may face isolation or lack of belonging (53). Further investigation is needed to understand the complex relationships between neighborhood connectedness on health care utilization and health outcomes among diverse AA patients.

Recent studies have shown a positive relationship between screening for and addressing HRSN and health care utilization, including cancer screening and treatment (82–85). In addition, lower rate of colorectal cancer screen are observed in other educated AA populations elsewhere, which we did not observe in our study. We did not observe significant differences in reported HRSN or neighborhood perceptions by cancer screening history in our exploratory analysis based on medical chart review. Some possible reasons for lack of significant associations include the high proportion of patients ever receiving and routinely receiving cancer screening at CAH, the temporality of HRSN and our cancer screening measures, and the smaller sample size of participants over age 50. The CAH has partnered with national and state level initiatives to focus on Hepatitis B screening (86) and colorectal cancer screening (ScreenNJ www.screennj.org), thus already high rates of cancer screening observed in our study may be a result of ongoing patient, provider, and clinic efforts. It will be important to monitor the impact of the COVID-19 pandemic on increased HRSN, delays in routine primary care visits, and delays in routine cancer screening moving forward, as recent data have shown large decreases in patient visits both nationally and within CAH between April and November 2020 (87–89). Furthermore, more research is needed on how HRSN may differentially impact disparities across stages of the cancer care continuum from screening to survivorship.

There are some limitations to our study that should be noted. First, we focus on a single primary care clinic that serves a high proportion of Chinese patients in a large suburban hospital system, contributing to a modest sample size. While our study population of largely immigrant, Chinese American patients may not be representative of the broad diversity of Chinese Americans or heterogeneous AAPI populations in New Jersey, it does provide important insight on HRSNs experienced by suburban Asian patients who are otherwise understudied but experience disparities in health and health care. In addition, our data are cross-sectional, precluding analyses of causality. We make numerous statistical comparisons, raising the possibility of finding significance by chance. We combined response categories in some HRSN measures due to small cell sizes and were not able to assess whether these edits impact the validity of the measures. Nevertheless, this study highlights the important need to focus on suburban AA patients, a largely understudied group, who may have social needs and access to support services distinct from their urban counterparts. Second, it is important to note, while these findings highlight the need to implement language appropriate health-related social needs screening tools for AA patients who otherwise would be omitted from clinic-based screening assessments, there is a need to address the heterogeneity of groups within Asian Americans and the community-specific factors that may impact health care utilization and outcomes. While we did find differences between Chinese-S and Chinese-T immigrant participants, we did not specifically compare acculturation using validated measures between these groups. The decision to compare across survey languages was informed by clinic providers and community partners. Per their experiences with the community, Chinese-T immigrants in NJ had largely immigrated earlier (many from Taiwan & Hong Kong) than many who were Chinese-S immigrants (mainland China) and thus many had more years in the US and might be more established and have fewer HSRN. This was seen in many of the measures but it did not hold true for transportation, highlighting vulnerabilities that come with older age and across groups. Third, although we made every effort to continue study recruitment using the same methods during the pandemic. Patients recruited during the pandemic (April 2020 and beyond) are likely those who could more likely overcome barriers and who felt safe from COVID exposure to access primary care again. Thus, the lack of change in HRSNs and neighborhood perceptions between pre-COVID-19 and during COVID-19 recruitment may be due to a bias from the differences in enabling factors among patients who were seen during the COVID-19 pandemic.

## Conclusions

This is one of few studies examining HRSN and related factors within AA populations in a health care setting. We observed higher than anticipated reports of HRSN, including high reports of interpersonal violence and housing needs among all Chinese participants, and low neighborhood perceptions among Chinese-S participants, suggesting the need to assess HRSN and the broader context of social determinants even among higher educated, suburban AA patients with health insurance. These study findings inform the need to adapt and augment HRSN data collection strategies to adequately address social needs and life course experiences for Asian language speaking patients within clinic settings. As efforts to address HRSN within clinical settings continue, including establishing systematic screening measures, implementation across settings, and policies to incentivize providers, it will be important to accurately measure the needs of all diverse racial/ethnic groups. It is also important to recognize and address the more upstream impacts of SDOH, including discrimination and structural racism, while efforts to focus on more downstream impacts of unmet health related social needs are ongoing (90). Institutional efforts to address implicit bias, structural racism and the other contributors to SDOH, as has been undertaken by the larger hospital system of CAH (91), are critical for confronting the many root issues and creating larger-scale change. As the impact of the COVID-19 pandemic on physical and mental health for racial/ethnic minority communities emerge, aspects of addressing unmet social needs will become even more important. Effectively addressing community-specific HRSN referral needs as well as more upstream social determinants of health that contribute to health and health disparities for Asian Americans will require multilevel strategies at the community, health system, and policy levels.

## Data Availability Statement

The datasets presented in this article are not readily available because Data cannot be shared publicly because of sensitive information collected from participants and the current protocol approved by the Saint Barnabas Medical Center Institutional Review Board. Requests to access the datasets should be directed to tsuijenn@usc.edu.

## Ethics Statement

This study was approved by the Rutgers Biomedical Health Sciences Institutional Review Board and the Saint Barnabas Medical Center Institutional Review Board. The patients/participants provided their written informed consent to participate in this study.

## Author Contributions

JT and SW planned the study. JT obtained funding, directed the data collection, analysis and interpretation, and led the manuscript writing. SW, RB, and JCa contributed to the study planning, funding acquisition, design and interpretation of study findings. AY, BA, and BX conducted the data collection and data acquisition. JCh conducted the data analysis and data revisions for the manuscript. AY, BA, and JCh contributed to the manuscript preparation. All authors contributed to the manuscript revisions, reviewed, and approved the final submitted manuscript.

## Conflict of Interest

The authors declare that the research was conducted in the absence of any commercial or financial relationships that could be construed as a potential conflict of interest.
